# Content validity of the Constructivist Learning in Higher Education Settings (CLHES) scale in the context of the flipped classroom in higher education

**DOI:** 10.1057/s41599-023-01754-3

**Published:** 2023-05-30

**Authors:** Turki Mesfer Alqahtani, Farrah Dina Yusop, Siti Hajar Halili

**Affiliations:** 1 Department of Curriculum and Instructional Technology, Faculty of Education, Universiti Malaya, 50603 Kuala Lumpur, Malaysia; 2 Department of Instructional Technology, Faculty of Education, Jazan University, Jazan, Saudi Arabia

**Keywords:** Education, Science, technology and society

## Abstract

During the COVID-19 pandemic, the flipped classroom (FC) approach has been a prominent teaching and learning strategy. Despite its popularity, few studies have been undertaken to effectively measure student learning experiences in an FC learning environment. The purpose of this study is to assess the content validity of the Constructivist Learning in Higher Education Settings (CLHES) scale, which is used to measure student learning experiences in a flipped classroom (FC) in the Saudi Arabian higher education environment. The content validity of the eight-dimension scale was examined using the three-tier methodology, including the content validity ratio (CVR) technique, based on the evaluations of selected experts in the field and factor analysis methodology. The results showed that 31 of the 32 items were accepted, with only one item being denied. The findings suggested that this instrument has a strong potential for usage as a valid scale to evaluate the quality of FC teaching and learning among higher education students.

## Introduction

The flipped classroom (FC) has appeared in recent years as a modern alternative to traditional learning methods (Seery, 2015). FC is defined as an educational practice that inverts the traditional course and homework components of a course (Bergmann and Sams, 2012). The FC, according to Bishop and Verleger (2013), is a successful teaching and learning method that helps student educational growth and improves learning experience and outcomes. As a result, the FC has gained popularity as a teaching approach that improves student abilities promotes active learning, and improves learning outcomes (Bishop and Verleger, 2013; Çakiroglu and Öztürk, 2017; Eichler and Peeples, 2016). Therefore, it is important to evaluate student learning experience in FC, especially in the higher education context, to provide evidence about the benefit of implementing the FC approach. Accordingly, the Constructivist Learning in Higher Education Settings (CLHES) scale was adapted to measure student learning experience in an FC environment. Alt (2014) has developed the CLHES scale that is used to evaluate constructivist activities in different educational environments such as seminars, distance learning, blended learning, and other environments. Therefore, it is crucial to validate each item in the instrument to ensure that it measures what it intends to measure through the validity test. In this study, content validity was utilized to conduct a validity test.

Content validity symbolizes the operations, which assures that the study’s instrument measures what it intends to measure, and the items are appropriate and represent the domain content (Frank-Stromberg and Olsen, 2004). Content validity aims at validating each item in the instrument, which represents variable dimensions (Miller et al., 2013). One of the ways of achieving content validity is to involve expert panels in a subject to consider the value and significance of items within an instrument. Where there are more proofs of content validity including expert opinions and evaluation, researcher confidence will be increased in the validity of the instruments used in the study (Johnson and Christensen, 2019).

Several methods were applied to measure the validity of different scales. Likewise, the content validity ratio (CVR) approach suggested by Lawshe (1975) is utilized in performing item validation. This method is usually used to measure and achieve the validity of the content of the research instruments in different fields of study such as healthcare, organizational development, education, and marketing research (Wilson et al., 2012) in addition to online learning studies (Kawachi, 2014; Mishra and Panda, 2007).

Lawshe (1975) recommended that the first step in CVR is to define the content domains of the questionnaire. Based on the recommendation, the classroom activities and student learning experience qualitatively and quantitatively were analyzed to define the content domains of the questionnaire (Alt, 2014). Consequently, Alt (2014) determined eight dimensions as the content domain of the questionnaire which are: *Construction of knowledge, Learning deeply, Authenticity, Perspective, Teacher–student interaction, Prior knowledge, Social interaction*, and *Cooperative dialog*. The eight dimensions are described as follows:

1. *Construction of knowledge* is defined as the opportunity given to students to raise questions about problems, investigate them, and look for potential explanations.
2. *Learning deeply* refers to the scope which gives a chance to students to explore a particular subject extensively.
3. *Authenticity* refers to giving related meanings to the concepts of learning and addressing events linked to academic issues and realistic topics.
4. *Prior knowledge* refers to linking the subject materials in a course with the topics of other courses.
5. The several *perspectives* refer to providing opinions from various viewpoints.
6. *Social interaction* refers to different learning exercises and learning activities.
7. *Teacher–student interaction* indicates the role of the instructor in the class which involves supporting and guiding students in the learning process.
8. *Cooperative dialog* refers to the discussion activities while conducting the lecture where learners can exchange original ideas and opinions.

To date, only a few studies focus on Middle Eastern students’ experiences with FC (e.g. Al-Harbi and Alshumaimeri, 2016; Alamri, 2019; Albalawi, 2018; AlJaser, 2017; Alrowais, 2014; Alsowat, 2016; Alzahrani, 2015a, 2015b; Elmaadaway, 2018), let alone studies that assess FC scale validity in Saudi Arabia’s higher education system especially using the Constructivist Learning in Higher Education Settings (CLHES) scale.

As a result, the purpose of this study is to add to the existing body of knowledge by assessing the content validity of the CLHES scale, which is used to assess student learning experiences in a flipped classroom in a Saudi Arabian higher education setting. The following research question is guiding this study: How do flipped classroom (FC) experts assess the content validity of the Constructivist Learning in Higher Education Settings (CLHES) scale in the context of Saudi higher education? The following section of this paper will summarize the literature on FC as well as student experiences in an FC learning environment.

## Review of literature

### Students’ learning experience in the flipped classroom

The FC is an excellent strategy for active learning and teaching that promotes educational development while improving student learning outcomes (Bishop and Verleger, 2013). The FC has lately evolved as a novel learning method distinct from the traditional approach (Al Mamun et al., 2022; Karabatak and Polat, 2020). In several educational institutions worldwide, FC has become popular, and instructors are becoming more aware of its importance (Gündüz and Akkoyunlu, 2019). The most important aspect of an FC environment is the retrieval of time that is normally spent teaching in the classroom (Bergmann and Sams, 2014). Flipped learning enables the educational system to shift from a teacher-centered learning environment to a learner-centered one (Awidi and Paynter, 2019; Gündüz and Akkoyunlu, 2019), where teachers present the course outside the classroom, which allows educators to dedicate class time to students working with peers, interacting with the learners or in groups to work on in-class activities (Halili and Zainuddin, 2015; Kovach, 2014; Kushairi and Ahmi, 2021).

Therefore, the nature of the FC approach is all about the student learning experience outside and inside the class, where students build their knowledge, learn intensively, interact with the teacher in the class, participate in learning activities, and collaborate with others. Because lectures and videos are offered outside of the classroom in FC, educators have more time for students individually, allowing for more time to be spent on discussions during class time. Hutchings and Quinney (2015) highlighted that instructors stated being excited about the FC and how it can maximize student class time. Also, teachers in an FC have felt they have a larger sense of educational freedom as they can spend longer time for learning activities through time in class (Gullen and Zimmerman, 2013; Milman, 2012). Further, teachers who utilize FC technique stressed that the best benefit of using FC for the first time in their teaching careers is that they have individual contact with each student during class time (Moore et al., 2014). According to Flumerfelt and Green (2013), the FC enables a great learning environment where learners can share knowledge with others, encouraging a significant personal achievement level for learners.

Moreover, teachers utilize different methods and techniques in the FC approach to provide self-directed learning and enhance the student learning experience such as discovery learning and problem-based learning (Namaziandost and Çakmak, 2020). In an FC the video lectures allow students to understand the material at their own speed (Brunsell and Horejsi, 2013; Goodwin and Miller, 2013) where they can control and repeat the videos with the ability to start and pause the videos many times based on individual need to absorb the materials, and providing lessons (Zhu et al., 2022). This makes learning more individualized for each student and may help them to enhance their academic performance according to their learning styles, including children with learning difficulties who would no longer fall behind in their studies and learning (Goodwin and Miller, 2013). In addition, Tucker (2012) emphasized that students enjoy the FC environment because they can make up for missing lessons or review lessons at their leisure.

### Flipped classroom instrument validation studies

With the expansion of the FC, there is a greater demand for scales to measure the success of the FC approach. Unfortunately, only a few research studies have been undertaken to create and evaluate appropriate instruments to measure FC, particularly the student learning experiences (e.g., Tang et al., 2021; Hidayat et al., 2021; Youhasan et al., 2020). Tang et al. (2021) conducted a study to verify a scale to assess students’ 21st-century competencies in the FC. Interest, Engagement, Collaboration, Creativity, and Self-regulation are the five dimensions of this measure. Another quite extensive study was conducted by Hidayat et al. (2021) that aimed at determining the required dimensions for a developed instrument for the FC. This study contains seven dimensions, namely professional educators, the shift in a learning culture, diversified seamless learning platforms, flexible environments, engaging and effective learning experiences, progressive networking activities, and intentional content. From a different perspective, another study by Youhasan et al. (2020) looked into another dimension of FC learning, which is the readiness to be engaged in FC learning from four dimensions which are personal, technological, pedagogical, and environmental readiness.

While all of these research have helped to provide the greatest instrument for measuring student learning experiences in higher education, the studies focused more on what happens inside the classroom. What is still lacking is the measuring of learning experiences that occur outside the classroom in addition to learning experiences that occur inside the classroom. This is because FC places a strong emphasis on self-learning and how students construct new information based on and/or integrate previous learning into new learning. As a result, there is a need for a tool that captures both students’ inside and outside-of-classroom learning experiences. Due to the emphasis on self- and independent learning in the FC method, such an instrument must also measure the many principles of constructivist learning techniques.

This study intends to build on past research by developing and validating a relevant instrument to assess higher education students’ learning experiences in an FC learning environment. The CLHES scale was chosen as the instrument to be validated for this purpose because its eight dimensions are regarded as comprehensive enough to cover both student learning experiences inside and outside the classrooms In this study, student learning experience refers to contact with the learning and teaching environment, and thus educational conduct is influenced by learning experience in the educational scenario (Shea and Bidjerano, 2010).

This study contributes to the existing literature by providing an instrument and guidelines for researchers and assessors to be used in assessing student learning experiences in the flipped classroom. Further, it provides a reference for researchers in adapting content validation measures in validating instruments to be used in assessing student learning experiences in the FC. Therefore, this study adds to the existing literature, including Alt and Raichel (2020) and Cochrane (2019), on the suitability of the scale to measure FC learning experiences in addition to problem-based learning, self-and peer assessment, and online education.

## Methods

**Fig. 1:**
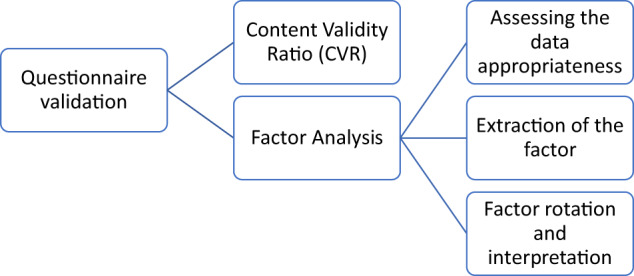
**Techniques used to validate the instrument**. Two techniques are used in this questionnaire validation processes, i.e., the Content Validity Ratio (CVR) technique and factor analysis.

In this present study, the quantitative approach was applied to validate the questionnaire using two techniques that are commonly used in questionnaire validation as in Fig. 1. These techniques that are used in questionnaire validation are the content validity ratio (CVR) technique and factor analysis. In this study, we will discuss in detail the steps involved in using these techniques to validate the questionnaire. The following sections will include more details regarding the instrument, the samples of this study, and the data collection procedure.

### Adapting the instrument

Due to the paucity of studies providing scales for measuring student learning experience in an FC environment, we widened our review to contain scales from studies with practical evidence that examined the student learning experience in blended learning and other e-learning approaches, with some modifications to wording to suit the FC context. In this study, the CLHES scale developed by Alt (2014) was adapted to measure student learning experience in the FC environment. The findings of the study revealed eight (8) dimensions as the domain construct of the questionnaire, namely: (1) *Construction of knowledge,* (2) *Learning deeply,* (3) *Authenticity,* (4) *Perspective,* (5) *Teacher–student interaction,* (6) *Prior knowledge,* (7) *Social interaction*, and (8) *Cooperative dialog* (Alt, 2014). The student learning experience in an FC environment is conceptualized in those eight dimensions. Details about the dimensions, assessment criteria, and the number of items are shown in Table 1.

**Table 1:** Dimensions, assessment criteria, and number of items.

Dimensions	Item no.	Assessment criteria (in this course,…)	No. of items
1. Construction of knowledge definition: The opportunities that are given to students to raise questions about problems, investigate them, and look for potential explanations.	1	“I was given opportunities to investigate real problems.”	5
	2	“I was given opportunities to raise questions about complex problems.”	
	3	“I was given opportunities to search for possible explanations for real problems.”	
	4	“I was asked to analyze data regarding a significant problem I have raised during this course.”	
	5	“I was asked to draw conclusions from a research work, in which I have participated”	
2. Learning deeply definition: The scope that gives a chance to students to explore a particular subject extensively.	6	“I have learned skills with which I can deeply explore a subject which is of interest to me”	4
	7	“I could examine in depth a major issue”	
	8	“I have focused on a central subject which I was required to deeply understand”	
	9	“I have learned how to investigate intensely a certain subject”	
3. Authenticity definition: Giving related meanings to the concepts of learning and addressing events linked to academic issues and realistic topics.	10	“This course addressed interesting situations in reality”	4
	11	“This course focused on giving relevant meaning to the learned concepts”	
	12	“This course addressed real life and interesting events”	
	13	“This course was rich with real-life examples that interest me”	
4. Perspective definition: Providing opinions from various viewpoints.	14	“Ideas were presented from several point of view”	4
	15	“I have learned about complex real issues”	
	16	“I have realized that the reality is complex and multi-dimensional”	
	17	“I had to question and criticize accepted ideas”	
5. Prior knowledge definition: Linking the subject materials in a course with the topics of other courses.	18	“This course dealt with subjects I have learned in other courses”	4
	19	“The subjects learned were related to prior knowledge I have gained”	
	20	“The things that I have learned have helped me understand issues which I have learned in other courses”	
	21	“The subjects were related to diverse contents of knowledge”	
6. Lecturer–student interaction definition: The role of the instructor in the class which involves supporting and guiding students in the learning process.	22	“The lecturer allowed me to think about my learning and how to improve it”	5
	23	“The lecturer considered my learning pace”	
	24	“I could set myself some learning goals”	
	25	“The lecturer encouraged me to think about my learning and ways to improve it”	
	26	“The lecturer made me think about the advantages and disadvantages of my learning”	
7. Social interaction definition: Different learning exercises and activities among learners.	27	“This course included a variety of learning activities with other students”	3
	28	“I was given opportunities to learn with other students”	
	29	“I could collaborate with other students”	
8. Cooperative dialog definition: The discussion activities while conducting the lecture where learners can exchange original ideas and opinions.	30	“Arguments and discussions were held”	3
	31	“It was possible to express original ideas”	
	32	“I could express my opinion, even when it was different from other students”	

Each dimension has several items, and there are 32 items in all (Table 1). These items were adapted to measure the student learning experience in the higher education FC context.

### Participants and data collection

After adapting the CLHES scale, the researchers proceeded to select expert panels to conduct CVR and then select students to collect data to conduct factor analysis. The data were collected in two phases: the first phase is to collect data from experts to conduct CVR, and the second phase is to collect data from students to conduct factor analysis.

*The first phase* is collecting data from experts; it is important to obtain the opinion of experts on the instrument to ensure that it is suitable for measuring the factors influencing the success of higher education FC in Saudi Arabia. The experts were purposefully chosen based on their expert knowledge and experience in instructional technology. In this study, purposive sampling was utilized, which is also known as judgmental or subjective sampling, to choose the experts.

A total of 24 experts were selected purposefully for this study, of which 18 experts have adopted the FC approach in their lectures at public universities in Saudi Arabia. Also, another six experts were FC researchers selected based on their publications in ResearchGate and the Web of Science database. The experts were contacted via cell phone and email to get their approval to participate and also to explain to them the procedures of the evaluation and the purpose of the study. They were asked to contribute by giving their opinions on an instrument validation questionnaire and answering the questionnaire. Out of the 24 experts, only 15 responses were received. Hence, the response rate is 63%. The backgrounds of all experts are included in Table 2.

**Table 2:** Backgrounds of all experts.

Expert	Gender	Institution	Academic position	Experience in questionnaire validation
Expert 1	Female	University of Jeddah	Associate Professor	6–10 Years
Expert 2	Male	King Khalid University	Assistant Professor	1–5 Years
Expert 3	Male	Shaqra University	Associate Professor	11–15 Years
Expert 4	Male	Jazan University	Associate Professor	6–10 Years
Expert 5	Female	King Abdulaziz University	Professor	16–20 Years
Expert 6	Male	Majmaah University	Lecturer	1–5 Years
Expert 7	Female	Princess Nora bint Abdul Rahman University	Assistant Professor	6–10 Years
Expert 8	Male	King Saud University	Professor	11–15 Years
Expert 9	Female	Taibah University	Assistant Professor	6–10 Years
Expert 10	Male	Imam Abdulrahman Bin Faisal University	Associate Professor	6–10 Years
Expert 11	Female	University of Jeddah	Associate Professor	11–15 Years
Expert 12	Male	Jazan University	Assistant Professor	1–5 Years
Expert 13	Male	Tabuk University	Assistant Professor	6–10 Years
Expert 14	Female	King Saud University	Associate Professor	11–15 Years
Expert 15	Male	University of Jeddah	Assistant Professor	1–5 Years

The second phase is collecting data from students to conduct factor analysis. After receiving the responses from the experts and validating all the questionnaire items, a pilot test was conducted on undergraduate students in Saudi Arabia universities. The questionnaire was distributed among 300 students who were chosen randomly. Some 200 responses were received, which were included in the pilot test of this study. Pallant (2016) indicated that at least 150 cases must be collected to ensure data suitability for factor analysis, whereas Sofroniou (1999) suggested that the sample size should be between 150 and 300 cases in factor analysis.

## Data analysis

The analysis was conducted in two steps: first, by assessing content validity using the content validity ratio (CVR) to estimate the items, and second by conducting factor analysis to identify underlying patterns and factors in the data. This approach allows for a more comprehensive understanding of the test validity and the relationship between its items.

### Estimating items using CVR

Lawshe (1975) proposed using the CVR to validate the items. The CVR represents the proportional level of the experts’ agreement in the panel and how many of them rated the item as “essential”. Thus, a three-point scale was used to measure the essentiality of each item in the instrument: (1 = not relevant, 2 = useful, but not essential, and 3 = essential). The CVR for every item is calculated based on the following formula:where *n_e* is the number of experts agreeing on an item as “essential” and *N* is the total number of expert panels. Table 3 presents the minimum values of CVR for different numbers of experts as mentioned by Lawshe (1975). In this study, the number of experts is made up of 15 members; therefore, the minimum value of CVR for the 15 experts is 0.49. This means that if the CVR value for an item exceeds 0.49, then the item will be kept because it is considered an important item. In contrast, the item will be removed from the scale when the value of CVR for the item is <0.49.

**Table 3:** Minimum values of CVR for different numbers of experts.

Number of experts	Minimum value
5	0.99
6	0.99
7	0.99
8	0.75
9	0.78
10	0.62
11	0.59
12	0.56
13	0.54
14	0.51
15	0.49
20	0.42
25	0.37
30	0.33
35	0.31
40	0.29

Source: Lawshe, C. H. (1975).

Based on the results in Table 3, if the CVR value for an item exceeds 0.49, then the item will be kept because it is considered an essential item. In contrast, the item will be removed from the scale when the CVR value for the item is below 0.49. Each item in the scale must be evaluated based on the CVR criteria. Each item must be validated by the expert panels whether the item is an ‘essential’, ‘useful but not necessary’, or ‘not necessary’ item to measure the domain construct of the questionnaire (Ayre and Scally, 2014; Cohen et al., 2010).

Data analysis was conducted after collecting the evaluations from the 15 experts using an online survey. The analysis was completed based on mathematical and statistical calculations using Microsoft Excel because the spreadsheet can be easily expanded by inserting columns or rows to add more items or experts, which makes it easy to calculate CVR for each item. The dataset and questionnaire of this study are available in a public data repository (Alqahtani et al., 2022a, 2022b).

### Factor analysis

The researchers utilized factor analysis to determine item validity in the flipped classroom questionnaire. Factor analysis is a method that makes a lower number of linear combinations of the primary variables in a way that retains the majority of the variability in the pattern of relationships (Pallant, 2016). Researchers in the early stage of research can employ the exploratory factor analysis (EFA) technique to gather information regarding the interrelationship between factors (Pallant, 2016). In the pilot test, the researchers applied the EFA technique to obtain data on the relationships between variables and to determine whether factor analysis was appropriate for each variable.

**Fig. 2:**
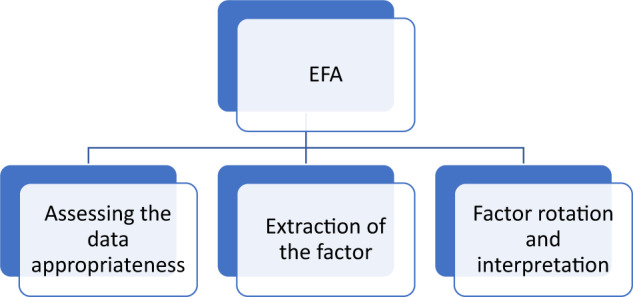
**Steps in exploratory factor analysis (EFA)**. Three steps were conducted in the exploratory factor analysis (EFA) technique: (1) assessing the appropriateness of the data for factor analysis; (2) extraction of the factor; and (3) factor rotation and interpretation.

For the EFA, the researchers applied three steps as shown in Fig. 2. They are: (1) assessing the appropriateness of the data for factor analysis, (2) extraction of the factor, and (3) factor rotation and interpretation (Pallant, 2016).

In the first step, Kaiser–Meyer–Olkin (KMO) and Bartlett’s Test of Sphericity were used to evaluate whether the data were suitable for factor analysis. These two tests are the most useful statistical measures that can assist in evaluating data factorability using the Statistical Packages for the Social Sciences (SPSS) (Pallant, 2016). Moreover, in the second step, information about the communities is provided, where the scree test, parallel analysis, and Kaiser’s criterion were all used. The commonalities relate to the information the researchers may obtain from each item in terms of how much variance each item has (Pallant, 2016). Pallant (2016) indicated that an item with a low value of <0.3 is unsuitable for combination with other items in its factor. As a result, all low-value items must be removed. The total variance explained will increase after removing items with a low value of commonality. In the last step, the Oblimin rotation was used (Pattern Matrix and Structure Matrix). In the Oblimin rotation, two loading tables were displayed: Pattern Matrix and Structure Matrix (Pallant, 2016). Oblimin rotation was used to extract all dimensions. Pattern Matrix presented information on the factor loading for each dimension. Based on that, the researchers employed factor analysis for the current study to determine the validity of the questionnaire items.

## Results

This section covers the results from measuring the content validity of the scale of student’s learning experience in higher education FC and the factor analysis results. As mentioned earlier, the adapted scale contains 32 items under eight construct domains for student learning experience in the FC. Therefore, the experts validated these items based on the results of the CVR, and subsequently, the researchers presented the results in Table 4. The number of experts who rated the item as an ‘essential’ out of 15 experts and the CVR value of each item are shown in Table 4.

**Table 4:** CVR values of the experts’ opinions and acceptance or rejection results.

Dimensions	Item no.	No. of experts who rated the item as an ‘essential’	CVR ≥ 0.49	Accept/Reject
1. Construction of knowledge	1	13	0.73	Accept
	2	14	0.87	Accept
	3	13	0.73	Accept
	4	13	0.73	Accept
	5	12	0.6	Accept
2. Learning deeply	6	13	0.73	Accept
	7	12	0.6	Accept
	8	14	0.87	Accept
	9	12	0.6	Accept
3. Authenticity	10	12	0.6	Accept
	11	14	0.87	Accept
	12	13	0.73	Accept
	13	13	0.73	Accept
4. Perspective	14	13	0.73	Accept
	15	13	0.73	Accept
	16	7	−0.07	Reject
	17	12	0.6	Accept
5. Prior knowledge	18	12	0.6	Accept
	19	14	0.87	Accept
	20	13	0.73	Accept
	21	13	0.73	Accept
6. Lecturer–student interaction	22	15	1	Accept
	23	13	0.73	Accept
	24	13	0.73	Accept
	25	13	0.73	Accept
	26	12	0.6	Accept
7. Social interaction	27	14	0.87	Accept
	28	12	0.6	Accept
	29	12	0.6	Accept
8. Cooperative dialog	30	13	0.73	Accept
	31	13	0.73	Accept
	32	13	0.73	Accept

**Fig. 3:**
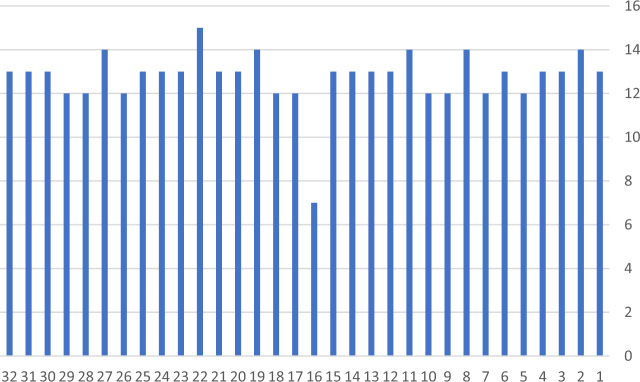
**Number of experts who rated the item as an** ‘**essential**’. The number of experts who rated each of the 32 items in the adapted questionnaire as ‘essential’.

The results in Table 4 revealed that the CVR value for 31 items is >0.49 and therefore these items were accepted. Only item 16 was rejected because the CVR value was below 0.49, which was (−0.07). Moreover, the majority of the experts suggested deleting item 16 because it was not understandable by respondents as shown in Fig. 3. The item read as “In this course, I have realized that reality is complex and multidimensional.”

In sum, the total number of accepted items based on the CVR results in this study is 31 items. All 31 items are able to measure student learning experience in the higher education FC setting.

Besides that, factor analysis was utilized to determine item validity in the flipped classroom questionnaire, where Kaiser–Meyer–Olkin (KMO) and Bartlett’s Test of Sphericity were used to evaluate whether the data were suitable for factor analysis. The KMO measures sampling adequacy, in which its index range ranges from 0 to 1 (Kaiser, 1974), with a minimum value of KMO for good factor analysis of 0.6 (Pallant, 2016). For Bartlett’s Test of Sphericity, the *p*-value must be <0.05 and significant (Bartlett, 1954; Pallant, 2016). If Bartlett’s Test of Sphericity is significant at a *p*-value of 0.05 or less, factor analysis is appropriate (Pallant, 2016; Tabachnick and Fidell, 2019). Table 5 displays the KMO and Bartlett’s Test results for the flipped classroom questionnaire.

**Table 5:** KMO and Bartlett’s test for the variable of flipped classroom.

Kaiser-Meyer-Olkin measure of sampling adequacy		0.904
Bartlett’s Test of Sphericity	Approximate Chi-square	3311.343
	Degree of freedom	465
	Significance	0.000*

* *p* < 0.05.

As shown in Table 5, the KMO was 0.904. Consequently, it is deemed an acceptable value because it exceeded 0.6, as Pallant (2016) and Kaiser (1974) suggested. Bartlett’s Test of Sphericity was likewise significant (*p* = 0.000) since the *p*-value was <0.05, as indicated by Bartlett (1954) and Pallant (2016).

The next step shows the commonalities findings. In this step, the commonalities findings are shown in Table 6.

**Table 6:** Results of The Pilot Study (EFA+ Communalities).

Item	Initial	Extraction
1	1.000	0.666
2	1.000	0.585
3	1.000	0.639
4	1.000	0.731
5	1.000	0.560
6	1.000	0.654
7	1.000	0.596
8	1.000	0.594
9	1.000	0.622
10	1.000	0.614
11	1.000	0.501
12	1.000	0.443
13	1.000	0.464
14	1.000	0.574
15	1.000	0.595
16	1.000	0.273
17	1.000	0.504
18	1.000	0.654
19	1.000	0.664
20	1.000	0.616
21	1.000	0.614
22	1.000	0.624
23	1.000	0.655
24	1.000	0.525
25	1.000	0.551
26	1.000	0.594
27	1.000	0.630
28	1.000	0.599
29	1.000	0.641
30	1.000	0.535
31	1.000	0.690
32	1.000	0.571

This pilot study’s questionnaire had 32 items with communality values over 0.5 for 31 items, which indicates that the results were acceptable with good results, except for item number 16 where the value was 0.273 which is below 0.30. According to Pallant (2016), any item with a low value of less than 0.30 is unsuitable for combination with other items in its factor. As a result, all low-value items must be removed.

Moreover, Kaiser’s criteria (or eigenvalue), scree test, and parallel analysis were utilized. A researcher should determine the suitable total variance when conducting the factor analysis. The variance must not be a maximum of 100% or at least 60% (Hair et al., 2022; Pallant, 2016). in the social sciences, however, a variance of 60% and, in some cases, even less is acceptable (Hair et al., 2022). In addition, Peterson (2000) conducted a meta-analysis study of variance accounted; this study found that the average percentage of accepted variance accounted for was 56.6% of the total variance. The following Table 7 displays Kaiser’s criteria (or eigenvalue) and parallel analysis values.

**Table 7:** Results of eigenvalue and parallel analysis for the FC questionnaire.

Dimensions	Initial eigenvalues			Extraction sums of squared loadings			Rotation sums of squared loadings
	Total	% of variance	Cumulative %	Total	% of variance	Cumulative %	Total
1	9.021	29.101	29.101	9.021	29.101	29.101	3.378
2	1.863	6.011	35.112	1.863	6.011	35.112	3.294
3	1.498	4.833	39.945	1.498	4.833	39.945	2.937
4	1.430	4.612	44.558	1.430	4.612	44.558	2.397
5	1.329	4.286	48.843	1.329	4.286	48.843	2.239
6	1.236	3.987	52.831	1.236	3.987	52.831	1.477
7	1.094	3.529	56.360	1.094	3.529	56.360	1.401
8	1.034	3.335	59.694	1.034	3.335	59.694	1.383
9	0.940	3.032	62.726				
10	0.904	2.915	65.641				
11	0.857	2.765	68.406				
12	0.828	2.670	71.077				
13	0.772	2.491	73.568				
14	0.698	2.252	75.820				
15	0.687	2.216	78.037				
16	0.643	2.073	80.109				
17	0.592	1.910	82.019				
18	0.559	1.804	83.823				
19	0.515	1.660	85.483				
20	0.495	1.598	87.081				
21	0.468	1.510	88.591				
22	0.428	1.382	89.972				
23	0.418	1.350	91.322				
24	0.409	1.319	92.641				
25	0.375	1.209	93.850				
26	0.372	1.199	95.049				
27	0.364	1.175	96.224				
28	0.329	1.061	97.284				
29	0.320	1.031	98.316				
30	0.268	0.866	99.181				
31	0.254	0.819	100.000				

**Fig. 4:**
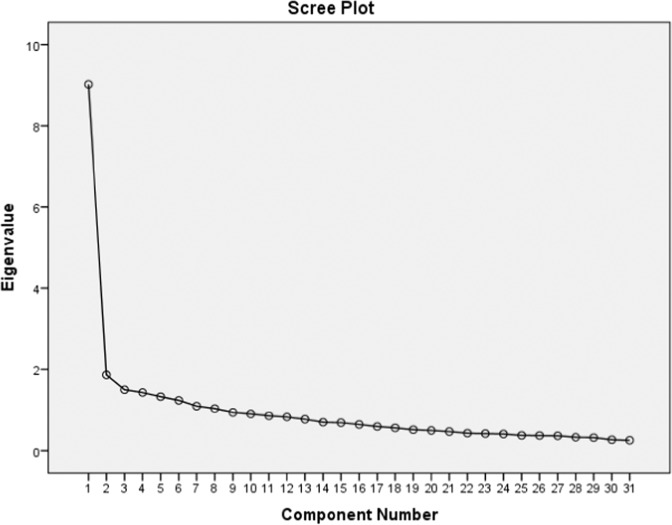
**The scree test results.** The scree test results show that all 31 items contribute significantly to the explained variance and should thus be retained.

As shown in Table 7 the flipped classroom questionnaire had eight eigenvalues larger than 1, accounting for 59.694% of the total variance. Furthermore, Yong and Pearce (2013) suggested using the scree test to determine the number of dimensions that must be retained. Figure 4 shows the scree test results.

In addition, the scree test is a method used in factor analysis to determine the number of dimensions or factors that should be retained in the analysis (Pallant, 2016). The scree test is based on the idea that as the number of factors increases, the amount of explained variance will also increase (Pallant, 2016). At some point, however, the increase in explained variance will level off, and any additional factors will have little impact. This point is called the “scree” in the scree plot, and the number of factors retained should be the number before the scree. Figure 4 shows the scree test results of this study.

As shown in Fig. 4, the scree plot shows that all 31 items are contributing significantly to the explained variance and therefore all of them should be kept.

Moreover, the final step of EFA is the Oblimin rotation; two loading Tables 8 and 9 are displayed. They are the Pattern Matrix and Structure Matrix (Pallant, 2016). In Oblimin rotation, the criterion or hypothesis is used to determine which values to accept or reject in the pattern matrix, and the structure matrix is based on the factor loadings (Pallant, 2016). Factor loadings, which are correlation coefficients between the factors and the variables, are used to assess the strength of the relationship between the factors and the variables (Pallant, 2016).

**Table 8:** Pattern matrix for eight dimensions.

Items	Dimensions							
	1	2	3	4	5	6	7	8
1	0.606							
2	0.530							
3	0.715							
4	0.916							
5	0.639							
6		0.750						
7		0.721						
8		0.592						
9		0.742						
10			0.439					
11			0.338					
12			0.369					
13			0.467					
14				0.380				
15				0.444				
16				0.514				
17					0.574			
18					0.755			
19					0.657			
20					0.650			
21						0.478		
22						0.552		
23						0.547		
24						0.701		
25						0.560		
26							0.502	
27							0.751	
28							0.804	
29								0.729
30								0.709
31								0.571

**Table 9:** Structure coefficient matrix for eight dimensions.

Items	Dimensions							
	1	2	3	4	5	6	7	8
1	0.67							
2	0.643							
3	0.756							
4	0.822							
5	0.677							
6		0.702						
7		0.736						
8		0.733						
9		0.741						
10			0.572					
11			0.507					
12			0.523					
13			0.556					
14				0.598				
15				0.621				
16				0.581				
17					0.578			
18					0.740			
19					0.706			
20					0.736			
21						0.668		
22						0.684		
23						0.539		
24						0.710		
25						0.541		
26							0.603	
27							0.686	
28							0.791	
29								0.726
30								0.706
31								0.629

In Oblimin rotation, Field (2013) recommended removing any items that have factor loadings less than 0.3 in the Pattern Matrix and Structure Matrix and also recommended each dimension should have at least three items with an acceptable loading score. Oblimin rotation was used to extract flipped classroom dimensions. The Pattern Matrix presented information on the factor loading for each variable, as shown in Table 8.

By applying the Pattern Matrix presented earlier in Table 8, all items had values above 0.3, which means the item should be kept. Further, items 1–5 were loaded on Dimension 1, items 6–9 on Dimension 2, items 10–13 on Dimension 3, items 14–16 on Dimension 4, items 17–20 on Dimension 5, items 21–25 on Dimension 6, items 26–28 on Dimension 7, and items 29–31 on Dimension 8.

Meanwhile, the Structure Matrix displayed the correlation between dimensions and variables, as presented in Table 9. The values provided in Table 9 were used to decide which items load on each of the dimensions.

Values displayed in the Structure Matrix earlier in Table 9, show that all item values were above 0.3, which means the item should be retained. Moreover, items 1–5 were loaded on Dimension 1, items 6–9 on Dimension 2, items 10–13 on Dimension 3, items 14–16 on Dimension 4, items 17–20 on Dimension 5, items 21–25 on Dimension 6, items 26–28 on Dimension 7, and items 29–31 on Dimension 8.

In sum, looking at the item loading on each dimension, each item’s shared qualities were identified. Dimension 1 (5 items) was labeled construction of knowledge, Dimension 2 (4 items) learning deeply, Dimension 3 (4 items) authenticity, Dimension 4 (3 items) perspective, Dimension 5 (4 items) teacher-student interaction, Dimension 6 (5 items) prior knowledge, Dimension 7 (3 items) social interaction, and Dimension 8 (3 items) cooperative dialog. As a result, the factor analysis for the flipped classroom is deemed appropriate.

## Contributions and implications

The main contribution of this study is the testament to the suitability and validity of the CLHES scale to measure student learning experiences within the FC learning environment, especially in the context of Saudi Arabia. The eight constructs of the scale are found to be compatible with the characteristics of FC such as the focus on learners rather than teachers and the principles of the constructivist learning approach in FC. This study will provide an instrument and guideline for researchers and assessors to use in assessing student learning experiences in the flipped classroom. Besides that, it provides a reference for researchers in adapting content validation measures in validating instruments to be used in assessing student learning experiences in the FC. The findings of this study illustrated that the CLHES scale by Alt (2014) is a useful instrument when applied to higher education in Saudi Arabia. In other words, this current study helps to fill the gap in the literature by providing empirical evidence of the suitability of utilizing the instrument in the flipped classroom approach, which was found to be efficient to implement in Saudi higher education. Therefore, this study adds to the existing literature on the suitability of the scale to measure FC learning experiences in addition to problem-based learning, self- and peer assessment (e.g., Alt and Raichel, 2020), and online education (e.g., Cochrane, 2019).

## Limitations and future research

This study is limited to the students who studied through the flipped classroom in the higher education sectors at various Saudi universities and excluded the learners in K-12. Furthermore, as this study was limited to one country, that means the findings should not be generalized because it is possible that different results may be found in different cultures.

Future research can expand upon this study in a few ways. One option would be to include a larger, more diverse sample of participants from various countries and educational settings to see if the findings can be replicated or if there are any cultural differences in the student learning experience in the FC approach and to generalize the findings and gain a more comprehensive understanding of student learning experiences in the FC. Additionally, future studies should attempt to evaluate the content validity of this scale based on levels of students by applying the same scale in the context of K-12 education.

## Conclusion and recommendations

The primary purpose of the current study was to evaluate the validity of the content and determine the suitability of the flipped classroom instrument. Additionally, this study is focused on measuring the content validity of students’ learning experiences in higher education using the FC approach. Originally, this study applied the CVR method to measure the content validity of students’ learning experiences based on 32 items. Furthermore, this study utilized factor analysis to determine the validity of the 32 items in the questionnaire. The findings, however, highlighted that only 31 out of 32 items are suitable.

Some recommendations for policymakers and designers in implementing, managing, and designing the FCs approach are made. One recommendation is ensuring that teachers are properly trained and supported in the use of technology and the flipped classroom model by holding more courses and workshops on the FC and how to implement it in classes. Another recommendation is fostering a culture of active learning and engagement in the classroom and providing opportunities for students to take ownership of their learning through activities such as discussion, collaboration, and project-based learning and provide resources to students for self-learning to ensure they can access material by themselves at their own pace.

In addition, monitoring and evaluating the flipped classroom approach and making adjustments as necessary to improve student outcomes are essential. Thus, higher education practitioners could utilize this provided instrument in this study to assess student learning experience in FC or any online environment. Besides, the higher education sector should provide the faculties and departments with specialists in instructional technology to assist teachers and lecturers in designing and creating learning videos, designing course materials, and helping them to publish all these materials and videos to students through educational platforms. Finally, engaging students in the adoption process, not only as users but as involvers in their opinions and decision-making is recommended. This involves discussing their opinions about developing the FC approach, their expectations, concerns, and the best ways of implementing this approach.

## Data Availability

The datasets generated during and/or analyzed during the current study are available in a public data repository, https://data.mendeley.com/datasets/5jdd74ggz8/6.
